# Exploring neural correlates of emotional and social listening with high-density diffuse optical tomography in the auditory cortex

**DOI:** 10.1038/s41598-026-49552-6

**Published:** 2026-08-26

**Authors:** Sruthi Srinivasan, Emilia Butters, Deepshikha Acharya, Ming Hung Hsu, Renee Timmers, Jennifer MacRitchie, Flavia Mancini, Gemma Bale

**Affiliations:** 1 Department of Engineering, University of Cambridge, Cambridge, UK; 2 Department of Psychiatry, University of Cambridge, Cambridge, UK; 3 Cambridge Institute for Music Therapy Research, Anglia Ruskin University, Cambridge, UK; 4 Department of Music, University of Sheffield, Sheffield, UK; 5 Department of Physics, University of Cambridge, Cambridge, UK

**Keywords:** Data processing, Auditory system, Cognitive neuroscience, Computational neuroscience, Social neuroscience

## Abstract

Listening to music impacts mood and stress levels, and may even improve cognition in older adulthood. How the brain responds to music may be different when the music evokes positive or negative emotions, when listening alone or alongside someone else. Hence, this study investigated whether the cortical, auditory responses to sad or happy music are different between solitary and social (dyadic) music listening. Using high-density diffuse optical tomography (HD-DOT), brain activity was measured in 88 volunteers, in a non-laboratory setting, while they first listened to music in a solitary, and then a dyadic, listening task. Significant differences in activation patterns were found between the two affective and social setting conditions, with the strongest differences in auditory response occurring between the social setting conditions. Additionally, comparable functional networks, namely the control, salience, and default mode networks, demonstrated higher connectivity in conditions where participants listened to music together, irrespective of musical affect. These networks also demonstrated large variations in inter-brain coherence between paired participants. This study validates HD-DOT’s effectiveness in relaxed environments and highlights its potential for large-scale data collection. Our results suggest that different social settings may influence brain functional metrics, with implications for how music can be used therapeutically across different health and care settings.

**Supplementary Information:**

The online version contains supplementary material available at https://doi.org/10.1038/s41598-026-49552-6.

## Introduction

Listening to music has long been shown to have significant impacts on mood and stress levels, due to music’s association with emotional arousal and valence^1–4^. When an individual listens to music, there are observable increases in cerebral blood flow across many brain areas and distributed networks^5^. Music is both enjoyed in solitary settings and in social contexts, and both situations may serve affective purposes. Additionally, some cognitive advantages associated with musical engagement in individuals of all ages have been documented^6^. However, as most neurological research is conducted on individuals in isolated settings, there is only a limited understanding of the neural processes in operation during social music listening^7,8^. How does social listening between pairs of listeners (dyads) compare in neurological activation patterns to individual listening? Does this difference vary with the affect of the music? In order to address these questions, we built a novel, functional near-infrared spectroscopy (fNIRS)-based experimental setup.

fNIRS is a non-invasive neuroimaging method that is highly portable and relatively robust to head motion, making it particularly well-suited to measuring brain activity in non-laboratory settings^9,10^. By using near-infrared light, relative changes in oxygenated (HbO) and deoxygenated (HbR) hemoglobin can be determined, which acts as an indirect measure of neural activity via the neurovascular coupling mechanism^11^. As opposed to traditional fNIRS, which typically uses a sparse array of light sources and detectors placed over the scalp, high-density diffuse optical tomography (HD-DOT) is a related neuroimaging modality that relies on the same underlying optical principles as fNIRS but allows for the reconstruction of more highly spatially resolved images of cortical activity using a high-density, multi-distance channel array and anatomical priors^12–14^. Given the image quality of HD-DOT, coupled with its relative insensitivity to motion and ease of use, it is a promising technology to study social interactions between individuals via hyperscanning.

Hyperscanning refers to the simultaneous measurement of brain activity across multiple individuals, and can be used to investigate neural synchrony, or the similarity in brain signals, between individuals^15,16^. fNIRS is one of the most prevalent modalities used in hyperscanning; the presence of brain-to-brain coupling, where the brain activity of two or more individuals is temporarily synchronized, has been previously observed in several fNIRS studies^17–20^. However, most hyperscanning experiments conducted in musical settings, across various modalities, focus on the dynamics of musical performance and joint music generation, with the subjects being musicians who were asked to play an instrument while their brain activity was recorded^16,21,22^. In contrast, very few hyperscanning studies have assessed the effects of passive music listening in a social context on the brain. For example, Osaka et al.^23^ used fNIRS to assess neural synchronization between pairs of participants who were asked to cooperatively hum or sing, both with and without eye contact, and found that regardless of social context, neural synchronization in the left interior frontal cortex significantly increased during cooperative music generation. In terms of hyperscanning studies conducted on participants while listening to music passively, Chabin et al.^24^ used EEG to measure neural coupling in attendees of an orchestral conducting competition and found that greater synchrony and higher self-reported pleasure scores were present in individuals when they were located physically closer to one another, which may point to mechanisms of emotional contagion in this setting. Emotional contagion, or the spontaneous mimicry of others’ emotional experiences, in group listening settings has also been demonstrated to be mediated by the degree of social bonding with others present in the group^25–27^. Other prior work involving hyperscanning in music listening has been conducted in the context of music therapy, on therapist-patient dyads^28^. However, many music therapy hyperscanning studies have focused on neural synchrony during music generation, rather than during passive listening^29,30^. Considering passive music listening has the advantage of being accessible to a broad population and is equally of interest for its potential positive effects on cognitive performance, its comparability and differences between listening in social and solitary contexts should be assessed^31^.

Thus, in this study, one of our primary aims was to assess the impact of social setting on neural synchrony, in the auditory cortex and immediately adjacent cortical areas, while paired participants were passively listening to music with different emotional affects. Recruiting participants from attendants of a music festival and collecting data outdoors on grounds close to the festival site provided a unique opportunity to record imaging data from a large number of participants. Previous work has established how increased interpersonal synchrony while listening to music can influence positive social feelings such as liking, connection, and affiliation^32–34^. We hypothesized that joint music listening is an important motivator for attendees at musical festivals and that the social context in which participants are placed influences their auditory brain response. Using fMRI, Koelsch et al.^35^ demonstrated that regions within the auditory cortex may contribute to the emotional processing of music, so we also expect to observe differences in brain activity across music stimuli evoking different emotions. Functional connectivity changes in the auditory cortex across different musical affects during listening have also been widely observed across a range of age groups^35,36^. While the wider influence of both social setting and musical affect on distributed brain networks may not be fully represented by looking at the auditory cortex, we can confidently expect to observe a neural response to music in this region, allowing us to assess other measures of interest, such as connectivity and synchrony^37,38^. Broadly, we hypothesized differences in patterns of activation, functional connectivity, and inter-brain coherence between the two social settings, and for these differences to be present in both affective conditions at varying degrees. In particular, we hypothesize that the effect of social context on music listening may be modulated by emotional affect: positive emotions, such as happiness, may be more social and thus music with a positive emotional affect may elicit greater brain activity in a social setting, while sadness may be a more individual emotion and elicit lower levels of activity overall.

## Results

Of the 160 datasets initially collected as part of this study, 100 datasets (62.5%) met the optical coupling criteria and were included in further analysis (see Methods). A further twelve datasets were excluded due to errors in data acquisition (7 datasets with unmarked experimental triggers delineating the start of a task block) and alcohol/substance screening (5 participants with >1 alcoholic drink in the five hours prior to taking part in the study). Thus, our final cohort was comprised of 88 adults (aged 41.2 ± 14.4 years). Of these 88 datasets, 50 of them belonged to a pair (25 pairs), meaning that both participants’ datasets were included in the paired analyses. Baseline demographic information about the full 160 participants can be found in Supplementary Table S1.

### Robustness of HD-DOT experimental setup

Despite allowing participants to move during data collection, we found the average percentage of the task period affected by motion to be minimal—approximately 4.82% of the task period suffered from motion artifacts (19.3s of total recording). We also found that within a total data collection period of ~25 hours (spread over four days), the ease of experimental setup enabled a data collection rate of ~3 dual scans per hour, demonstrating the portability and robustness of HD-DOT as a brain imaging method, particularly in non-laboratory settings. However, 60 of the 160 datasets (37.5%) originally collected were excluded due to poor optical coupling to the scalp (see Methods).

### Differences in activation across conditions

**Fig. 1:**
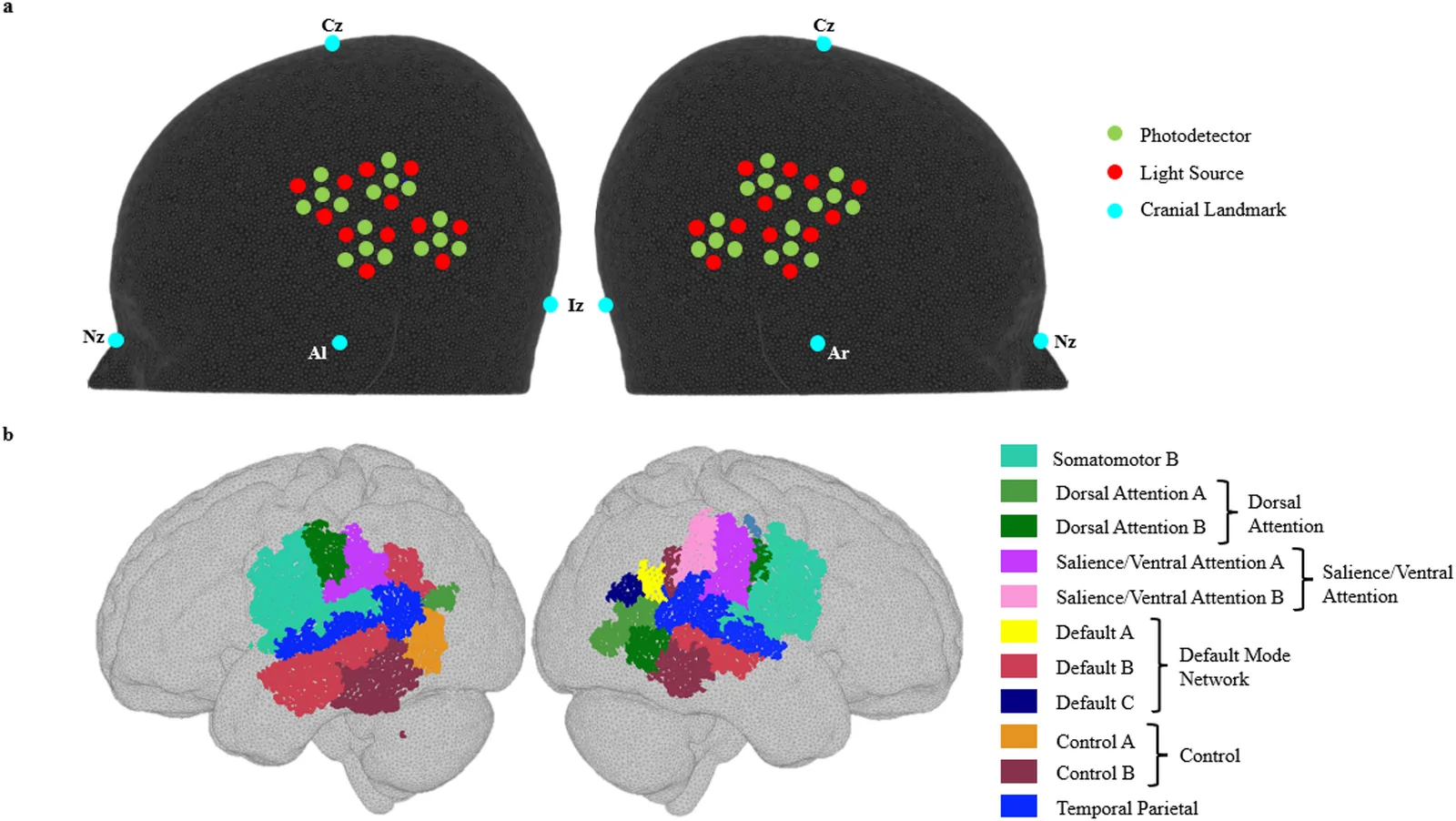
HD-DOT optode array layout and cortical array sensitivity. (**a**) Lateral view of light source (red circles) and photodetector (green circles) locations following registration to a standard head model with cranial landmarks (light blue circles) shown for reference. (**b**) Parcellated region of the cortical surface to which the HD-DOT optode array in use was sensitive. The different colors represent the respective functional networks. Certain adjacent networks were grouped together, as shown in the key, in subsequent analyses.

**Fig. 2:**
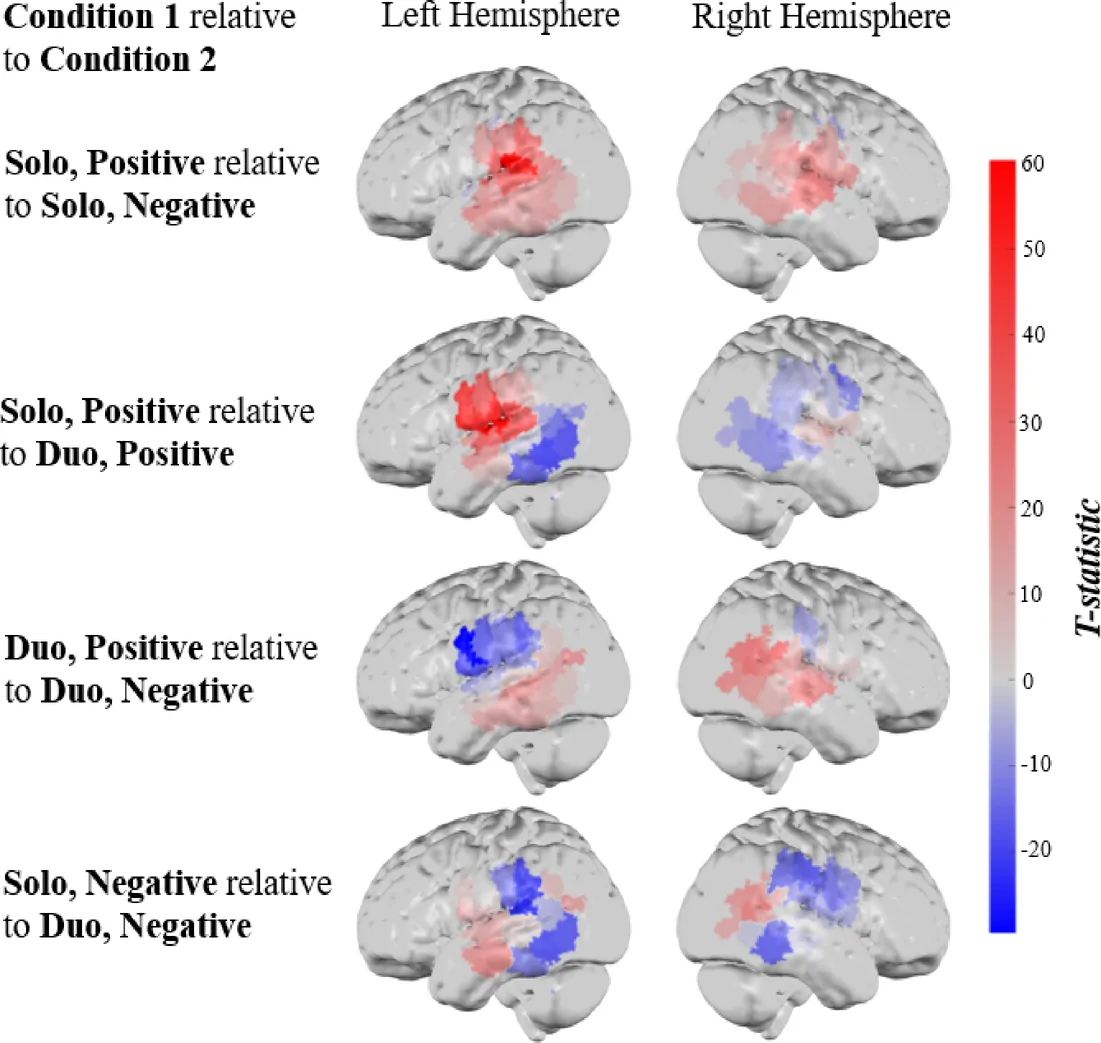
T-statistic maps comparing group-level (*n* = 88) HbO concentration changes for the four conditions. Values displayed indicate significant differences between parcels at the *p* < 0.05 significance level following FDR correction. Positive values of the T-statistic (shown in red) indicate regions where condition 1 demonstrated greater activity than condition 2, while negative values of the T-statistic (shown in blue) indicate regions where condition 2 demonstrated greater activity than condition 1.

Our HD-DOT optode layout (Fig. 1a) was sensitive to 54 bilateral parcels across 11 functional networks (Fig. 1b) when using a 400-parcel parcellation atlas (given that there are an estimated 300-400 distinct neocortical areas in the human brain)^39,40^. Any subsequent mention of functional networks refers *only* to neural activity observed within the recording regions shown in Fig. 1b and should not be attributed to the wider distributed brain networks. At the group-level, higher levels of activity, defined as a relative increase in HbO concentration from baseline, were observed in the solo listening case overall, with the solo, positive condition demonstrating the highest level of activity out of all conditions. Higher levels of activity, particularly in the right hemispheric default mode network, were also observed when listening to the positive affect music across both the solo and duo cases (see Supplementary Fig. S1). Figure 2 shows the cortical T-statistic maps for changes in HbO across the task period for one condition with respect to another. Higher (absolute) values of the T-statistic were observed in the control networks of the left hemisphere and the somatomotor B and salience networks of the right hemisphere, for both the duo, positive and duo, negative conditions (Fig. 2; rows 2 and 4). For all analyses, it is important to note that Task 1 refers to the solo condition and Task 2 refers to the duo condition across all participants.

### Functional connectivity

**Fig. 3:**
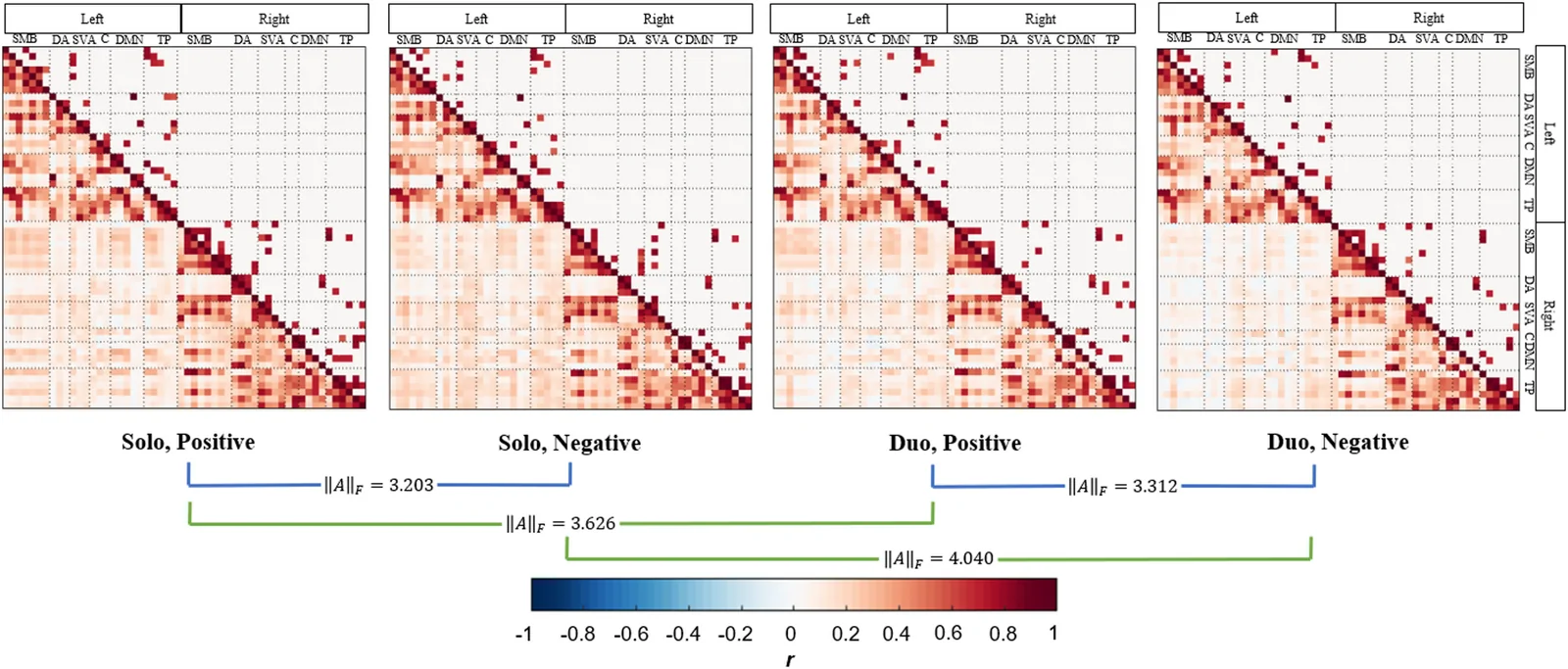
Static connectivity matrices (plots of Pearson’s *r*) for each of the four conditions, averaged over *n* = 88 participants. In each case, the lower triangular region is the full connectivity matrix representing the correlation between each pair of parcels (HbO), and the upper triangular region includes only parcels with significant correlations following FDR correction at the *p* < 0.05 level. Frobenius distance, ${\Vert A\Vert }_{F}$, between each pair is shown. Blue brackets delineate distance metrics between affective conditions, and green brackets delineate distance metrics between social setting conditions. Network abbreviations: Somatomotor B (SMB), Dorsal Attention (DA), Salience/Ventral Attention (SVA), Control (C), Default Mode Network (DMN), Temporal Parietal (TP).

Static functional connectivity was assessed per-participant over the block-averaged task period (30s), and then group averaged, as seen in Fig. 3. The solo, positive condition demonstrated the greatest number of significant connections (85 out of 1431 possible connections), consistent with the larger magnitude of activation observed for this condition. To assess the difference between each pair of connectivity matrices, Frobenius distance, ${\Vert A\Vert }_{F}$, was calculated. Larger differences in connectivity were seen for the solo, positive vs duo, positive condition (${\Vert A\Vert }_{F}=3.626$) and the solo, negative vs duo, negative condition (${\Vert A\Vert }_{F}=4.040$), as compared to the Frobenius distances between the different music types across both social listening cases. In all conditions, greater connectivity, defined here as a larger number of significant connections, was observed in the right hemisphere. In all cases, Frobenius distances were found to be statistically significant at the *p* < 0.05 significance level.

**Fig. 4:**
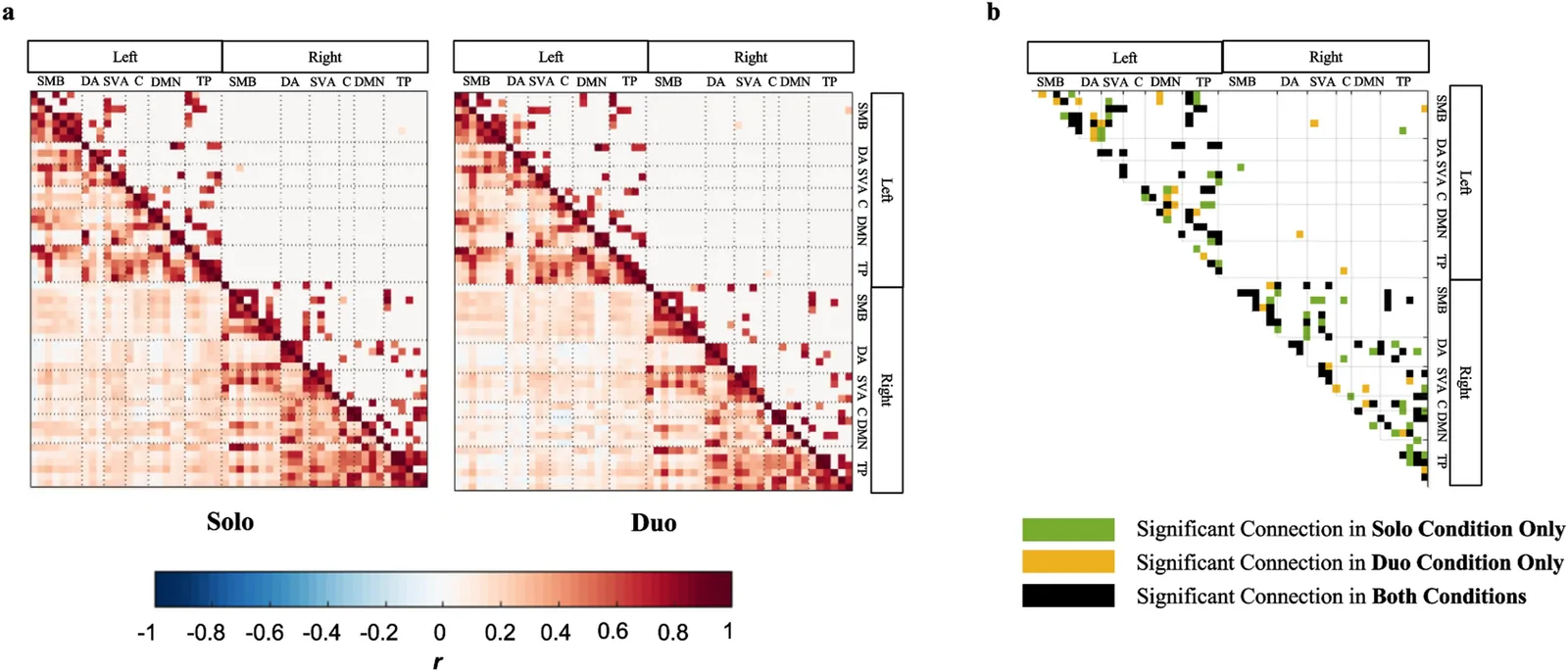
Static connectivity matrices (plots of Pearson’s *r*) for solo and duo listening conditions across both music types, averaged over only the subset (*n* = 44) of participants that were listening to the same set of music in tasks 1 and 2. (**a**) Functional connectivity for the solo and duo listening conditions. In each case, the lower triangular region is the full connectivity matrix representing the correlation between each pair of parcels (HbO), and the upper triangular region includes only parcels with significant correlations following FDR correction at the *p* < 0.05 level. (**b**) Upper triangular matrix depicting significant parcel correlation differences between solo and duo cases, where green squares represent connections that were significant only in the solo condition, yellow squares represent connections that were significant only in the duo condition, and black squares represent connections that were significant in both cases. Network abbreviations: Somatomotor B (SMB), Dorsal Attention (DA), Salience/Ventral Attention (SVA), Control (C), Default Mode Network (DMN), Temporal Parietal (TP).

We further explored differences in static connectivity between the solo and duo conditions (Fig. 4), exclusively for those participants that listened to the same set of music across Tasks 1 and 2 (Participant A in each paired recording, *n* = 44). This was done to eliminate any differences caused by listening to a new set of music in both the solo and duo conditions and to isolate differences in connectivity between the two social listening conditions. As seen in Fig. 4a, we found that there was a greater number of significant connections at the group level when looking at this subset of participants for both the solo and duo conditions (141 and 121 significant connections, respectively). In contrast, the average number of significant connections when looking at the entire participant cohort, across all four conditions, was 73.25 ± 7.85 (roughly half of the number of significant connections for this subset), indicating that the proportion of significant connections increased when isolating the effect of social setting. We also found that larger differences in connectivity, as measured using Frobenius distance, ${\Vert A\Vert }_{F}$, were found between the solo and duo listening conditions for the participant subset (${\Vert A\Vert }_{F}=3.824$), as compared to the entire participant cohort (${\Vert A\Vert }_{F}=3.111$). Once again, Frobenius distances were found to be statistically significant for both comparisons, at the *p* < 0.05 significance level. Additionally, we found that there was significant inter-hemispheric connectivity, particularly in the duo condition, which was not observed when looking at the entire participant cohort (Fig. 4b). A greater number of significant intra-hemispheric connections was observed in the right hemisphere for the solo conditions, as compared to the duo conditions, across all functional networks, while both intra- and inter-network connectivity in the left hemispheric default mode network increased for the duo conditions.

### Wavelet coherence

**Fig. 5:**
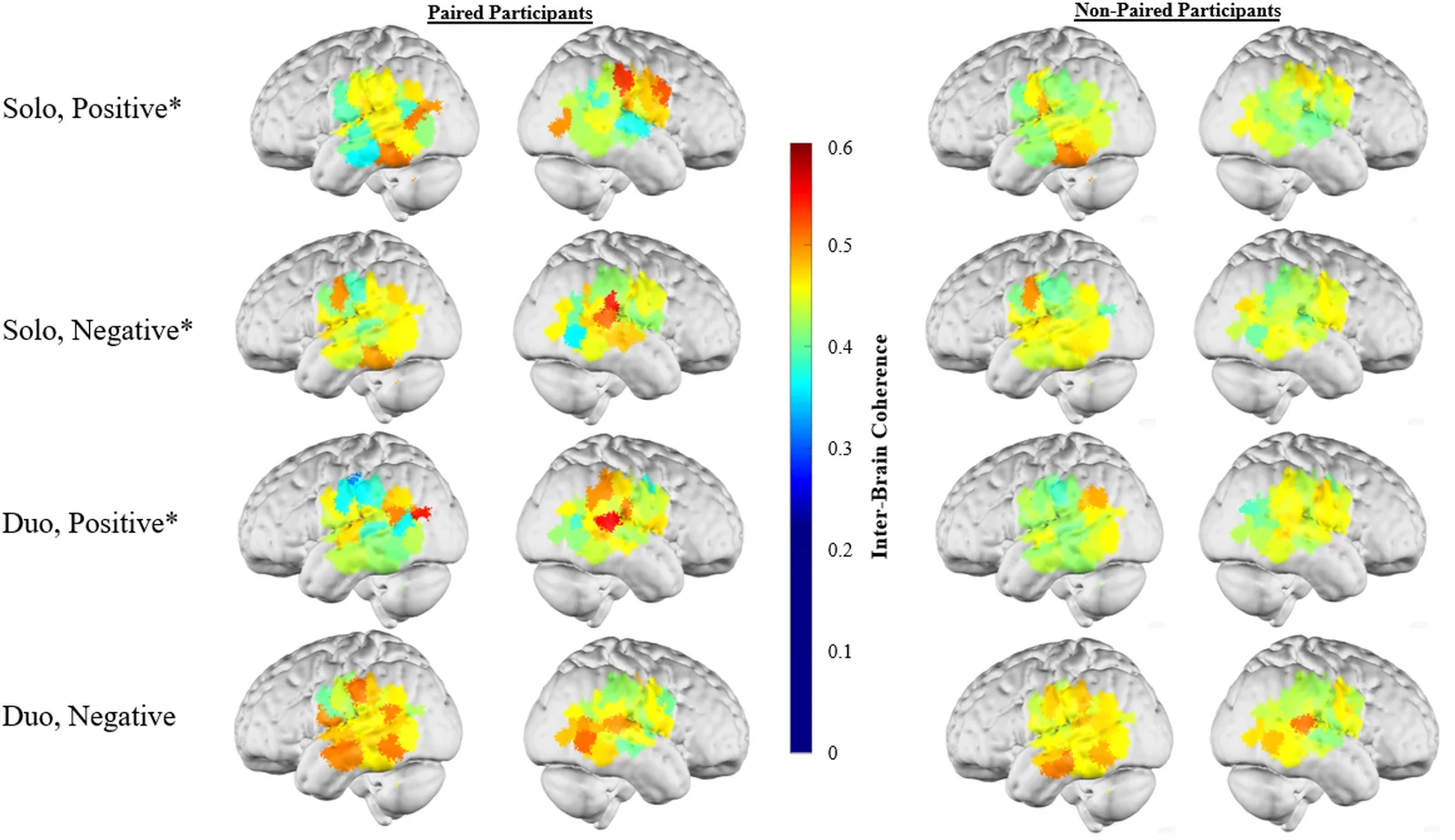
Inter-brain coherence between HbO signals for each of the four conditions, group averaged over *n* = 25 pairs (50 participants). Values displayed in the first two columns indicate mean wavelet coherence between each matching parcel across paired participants, while those in the last two columns indicate mean wavelet coherence between each matching parcel across non-paired participants. Color bar indicates degree of inter-brain coherence, as calculated using wavelet coherence within a low-frequency band (coherence values can range between 0 and 1). Conditions marked with an asterisk (*) indicate conditions for which significant differences in mean coherence per-parcel were found between all paired and non-paired participants following FDR correction at the *p* < 0.05 level.

Coherence between participants’ HbO brain responses was investigated to evaluate the degree to which inter-brain synchrony was present during the task period (Fig. 5). To ensure that the observed regional differences in inter-brain coherence were a function of the true participant pairings, we also assessed coherence between every non-pair (pairs comprised of two participants who did not perform the original experiment concurrently but who listened to the same music excerpts as each other) for each of the four conditions. In the solo and positive, solo and negative, and duo and positive conditions, significant differences in mean coherence per-parcel were found between all paired and non-paired participants following FDR correction at the *p* < 0.05 level. By contrast, significant differences in mean coherence were found for only 41% of all paired and non-paired participants in the duo and negative condition (FDR-corrected, *p* < 0.05). Euclidean distance between vectors containing the mean coherence values per-parcel for paired and non-paired participants was lowest for the duo and negative condition (0.2229), as compared to the other conditions (0.3094 for solo and positive, 0.3370 for solo and negative, and 0.3492 for duo and positive), indicating that coherence varied the least between paired and non-paired participants, at the group-level, in the duo, negative condition.

Larger variations in inter-brain coherence were seen among the paired participants, with distinct regions of increased and decreased coherence, when compared to non-paired participants, for each of the four conditions. In particular, greater coherence (> 0.55) was observed in the right hemispheric default mode network for the duo, positive condition, as compared to all other conditions, while greater coherence was observed in the bilateral dorsal attention network for the solo, positive condition (> 0.5). In all four conditions, but primarily in the solo and positive, solo and negative, and duo and positive conditions, regions of decreased coherence were found in the left hemisphere. In particular, reduced coherence (< 0.4) was observed in the left hemispheric default mode network for the solo, positive condition and in the left hemispheric dorsal attention and salience networks for the duo, positive condition (< 0.35). There was also a reduction in coherence in the left hemispheric dorsal attention network for the solo, negative condition. To assess which conditions elicited similar coherence patterns to each other, the Euclidean distance between vectors containing the HbO coherence values at each parcel was determined for each pair of conditions. The largest difference in coherence across the recording region for paired participants occurred between the solo, positive and duo, positive conditions (0.448), while the smallest difference in coherence occurred between the solo, negative and duo, negative conditions (0.329), indicating that differences in inter-brain coherence across social listening setting were most pronounced when pairs were listening to the positive affect music.

### Participant self-reported responses

To assess whether the selected music pieces evoked the emotion they were intended to (e.g., ‘happy’ music evokes happiness over any other emotion), group-level statistics were calculated based on the self-reported responses provided by participants on their post-task questionnaires. Self-reported numerical responses ranged from 0 to 4, with 0 corresponding to a qualitative response of ‘not at all’ and 4 corresponding to a qualitative response of ‘very much’. As seen in Table 1, the positive affect music evoked happiness to the largest degree (solo score: 2.81 ± 0.99, duo score: 2.86 ± 1.02), closely followed by excitement, in both the solo and duo listening tasks. The negative affect music evoked calmness to the largest degree (solo score: 2.34 ± 1.21, duo score: 2.31 ± 1.14), with feelings of sadness being ranked more than a full point lower on average than its counterpart in response to the positive affect music.

**Table 1:** Group distribution of participant responses ranking emotional response, familiarity, and liking after both the solo and duo listening tasks.

Question		Solo listening	Duo listening
		Mean ± Standard deviation	Mean ± Standard deviation
Emotion (‘Happy’ Music)	Excited	2.29 ± 1.19	2.49 ± 1.28
	Calm	1.94 ± 1.10	1.82 ± 1.16
	Scared	0.17 ± 0.49	0.14 ± 0.49
	Sad*	0.38 ± 0.91	0.23 ± 0.62
	Happy	2.81 ± 0.99	2.86 ± 1.02
Emotion (‘Sad’ Music)	Excited	1.03 ± 1.19	0.94 ± 1.06
	Calm	2.34 ± 1.21	2.31 ± 1.14
	Scared	0.56 ± 0.85	0.44 ± 0.82
	Sad	1.68 ± 1.04	1.68 ± 1.23
	Happy	1.17 ± 1.15	1.32 ± 1.07
Liking: ‘Happy’ Music		2.63 ± 1.10	2.55 ± 1.11
Liking: ‘Sad’ Music*		2.61 ± 1.13	2.38 ± 1.19
Connection to the Music		2.48 ± 1.18	2.34 ± 1.12
Connection to Other Listener		N/A	2.56 ± 1.20

Numerical responses ranged from 0 to 4, with 0 corresponding to a qualitative response of ‘not at all’ and 4 corresponding to a qualitative response of ‘very much’. Rows marked with an asterisk (*) indicate questions for which significant differences in responses were found between the solo and duo listening conditions at the *p* < 0.05 level, following Bonferroni correction.

Spearman’s rank order correlation revealed that there was no correlation between relationship length (in years) and the level of felt connection in the duo listening task (*ρ* = − 0.021, *n* = 88, *p* = 0.35). However, a Kruskal-Wallis one-way ANOVA showed significant differences between relationship type groups in terms of the average feeling of connection to the paired listener (H(3) = 16.19, *p* = 0.0028). Representative statistics from these relationship subgroups can be found in Table 2, with the highest average felt connection occurring in pairs that were either married or partners (3.05 ± 1.06) and the lowest average felt connection occurring in pairs that were strangers (1.70 ± 1.06), as expected.

**Table 2:** Distribution of participant responses ranking felt connection to the other listener during the duo listening task, separated into subgroups based on type of relationship between each pair.

Type of relationship (Group Size)	Connection to other listener (Mean ± Standard Deviation)
Friend (*n* = 29)	2.21 ± 1.21
Family (*n* = 7)	2.29 ± 1.11
Married/Partner (*n* = 42)	3.05 ± 1.06
Stranger (*n* = 10)	1.70 ± 1.06

Numerical responses ranged from 0 to 4, with 0 corresponding to a qualitative response of ‘not at all’ and 4 corresponding to a qualitative response of ‘very much’.

Spearman’s rank order correlation found a weak positive correlation between mean level of felt connection in the duo listening task and difference between mean paired and unpaired coherence in the duo listening task (*ρ* = 0.2174, *n* = 25, *p* < 0.05), indicating that larger positive differences in paired and unpaired coherence values were slightly associated with greater levels of felt connection between pairs.

## Discussion

As part of this study, we were able to assess the degree to which social setting plays a role in functional brain responses when passively listening to music of two different emotional affects. Significant differences in spatial activation patterns between each of the four conditions were found, identifying unique networks of activity for different affective conditions and social listening settings. We expected to observe greater activation for the positive affect music, and in general, we found that the largest magnitude of activation occurred in the solo, positive condition. This may, in part, be related to the stronger induction of emotion in the positive affect music condition than in the negative affect music condition, which evoked mild feelings of calmness and weak feelings of sadness.

Using film music to evoke emotion is common practice in experimental studies. Nevertheless, the present study cohort demonstrated large variations in the degree to which the intended emotions were felt, as seen in Table 1 (SD of >1 point in both the solo and duo listening conditions)^41^. In fact, previous work has demonstrated the subjectivity of music in eliciting specific emotions, as music is often tied to individual memories and other subject-specific features^42,43^. While the aim of this study was not to assess whether generalized music pieces can evoke standard emotions across individuals, it is possible that individual participants’ musical preferences and level of musical experience played a strong role in the degree to which the intended emotion was felt during passive listening, as has been demonstrated previously^44–46^. However, we found no difference in the level of brain activation observed in participants who reported familiarity with the music pieces in the experiment compared to those who were previously unfamiliar with the music pieces, across all four conditions. This is consistent with results from Fuentes-Sánchez et al.^44^, where it was found that familiarity did not play a significant role in music-induced emotions.

We also identified differences in static functional connectivity across all four conditions. Across all conditions, we observed greater connectivity in the right hemisphere compared to the left hemisphere, particularly within the temporal parietal network, but also between the temporal parietal network and other functional networks covered by the recording region. This is consistent with previous work identifying right hemispheric lateralization of tonal processing in music^47–49^. Larger differences in connectivity were observed between the two social settings, as opposed to the two affective conditions, which may imply that the social setting played a larger role on brain connectivity in the region of interest. Interestingly, we also observed heightened inter-network connectivity between the left hemispheric default mode network and other functional networks in the duo listening condition, compared to the solo listening condition (Fig. 4). One possible explanation for this may be that in the duo listening case, individuals paid less focused attention to the music itself and more attention to their study partner or surroundings. Indeed, the default mode network demonstrates higher activity and connectivity when less focused attention is paid to a task and is often implicated in the experience of mind wandering^50–52^. Given that participants always completed the solo condition first, another possible explanation for these connectivity differences may be that participants paid more attention to the music stimulus when first listening to it (solo condition), and subsequently focused on other stimuli, thus allowing their mind to wander more, when the music was later repeated (duo condition). This may also explain why participants felt slightly less connection on average to the music in the duo listening case, compared to the solo listening case.

Finally, inter-brain coherence was assessed for each pair of participants. We found that different functional networks demonstrated varying levels of synchrony between paired participants across each of the four conditions. Consistent with our connectivity results, greater coherence was also observed in the default mode network for the duo, positive condition, compared to all other conditions. Significant differences in coherence between paired and non-paired participants were found in the left hemispheric dorsal attention and salience networks for the duo listening cases, which may again point to a shift away from attention paid to the music itself and toward the study partner. The salience network is known to play a role in attention switching, while the dorsal attention network is implicated in attentional control^53–55^. Broadly, the observed increased coherence in duo listening conditions, and the weak positive association found between level of felt connection and difference in paired and unpaired coherence values, may be partially attributed to emotional contagion in paired participants, whereby a similar emotional experience is shared across individuals, in line with previous findings of emotional contagion characterized by increased inter-brain synchrony^56,57^.

To our knowledge, this is the largest adult HD-DOT dataset collected to date. In this study, we aimed to validate the use of HD-DOT in environments far removed from the typical clinical setting in which it has been traditionally applied. The robustness of the hemodynamic responses, despite the uncontrolled environment, demonstrates that HD-DOT is an effective neuroimaging method for performing non-laboratory studies. We found that, despite allowing participants to move during the task, our mean motion burden across all recordings was comparable to previous findings of motion burden in seated, laboratory-based experiments where participants were explicitly instructed not to move^58^. This provides further evidence as to the relative insensitivity of HD-DOT to motion, when compared to other brain imaging methods such as functional magnetic resonance imaging.

However, there are several limitations of the current work to consider. For one, our study design did not control for adaptation or habituation effects, as every participant was instructed to first listen to music alone, followed by a period of listening to music together. This was done for practical reasons, such that the different music types (happy/sad) could be investigated as independent variables in task 1 since participants would not have heard the music previously, but regardless, the significance of task order and affective stimulus presentation order in this study cannot be ruled out and future studies should employ counterbalancing measures to mitigate this. Additionally, a fundamental limitation with both fNIRS and HD-DOT is that hemodynamic activity can only be recorded from shallow cortical regions, while activity in deeper cortical and subcortical structures cannot be reliably imaged using these methods due to the exponential increase in light attenuation^59^. These deeper brain structures are often implicated in studies of music-induced emotion because they consistently demonstrate activity changes in response to music^60–62^. While we selected the auditory cortex and surrounding areas to ensure a robust hemodynamic response, as we knew these areas would be active in an auditory paradigm, we recognize that other regions of interest, particularly the prefrontal cortex, may have been a better choice to interrogate an emotional response to music^63^. Furthermore, subject-specific differences in HD-DOT array placement and head shape, both of which have been shown to have significant impacts on what brain regions are imaged, were not accounted for in the present work^64^. While photogrammetric information was collected for all participants, with the aim of determining subject-specific array locations for each participant, errors in a number of video recordings necessitated that identical array placement was assumed for all included participants. However, the error introduced using this assumption was mitigated by using a lower resolution parcellation atlas (400 parcels) and focusing on broad functional networks, rather than individual parcels. Finally, another inherent limitation may be that the music festival attendees were not representative of the general populace: the attendees choosing to participate in the study may already experience either a greater degree of social bonding or affinity for different affects of music through listening, as compared to non-attendees, which may introduce a degree of self-selection bias. Self-reporting bias, particularly given the reliance on questionnaire-based participant screening, may have resulted in the underreporting of alcohol or substance use. Given the experimental setup, formal testing for alcohol or substance use was not feasible; however, social desirability bias in participants’ responses may have been somewhat mitigated, as data collection occurred during the daytime in a relaxed environment. While our results can be enhanced in several ways, we believe that this work serves to validate the use of both HD-DOT and a paired auditory paradigm in a relaxed setting, while illuminating potential neural correlates of social music listening. In addition, the existence of such a large-scale dataset presents numerous opportunities for big data and machine learning analyses of HD-DOT data.

## Conclusion

This study demonstrates the successful use of HD-DOT to perform a paired auditory experiment across a large population, in order to assess the degree to which auditory responses to passive music listening are dependent on social setting. Our use of a portable HD-DOT system demonstrates the feasibility and relative ease of recording functional data in non-laboratory settings, underscoring the potential for more complex, naturalistic studies to be carried out in the future. As part of this work, we were able to identify specific functional networks that are active depending on the type of music being listened to, which suggests that different emotional affects were encoded in the fNIRS signals originating from the auditory cortex. We were also able to identify differences in static connectivity and brain-to-brain synchrony between different social listening settings, suggesting differential network recruitment when listening to music alone or around others. Taken together, these findings highlight the promise of portable HD-DOT systems for investigating how social context shapes auditory neural processing in ecologically valid environments.

## Methods

### Participants

160 adult participants took part in this study (aged 38.2 ± 13.7 years). All participants were attendees at the 2022 Green Man music and science festival in Wales. Demographic information as well as information on prior musical experience was collected from all participants. Additionally, information on alcohol and other substance consumption prior to partaking in the study was collected to allow for participant screening and exclusion. To collect data from a large sample of festivalgoers, all individuals aged 16+ were considered for inclusion in this study. All participants were recruited, and all data collection was carried out, at the festival over a period of four days. In this study, participant selection was not randomized. Instead, recruited participants were all festivalgoers who volunteered to take part in the study, and data from all participants aged 16+ were collected. Exclusion was based on whether participants had >1 alcoholic drink in the five hours prior to taking part in the study or had specified any other substances taken. All participants provided written informed consent prior to participating in the study. This study was approved on 14th July 2022 by the ethics committee within the Department of Engineering at the University of Cambridge. The research was performed in accordance with relevant regulations.

### Data acquisition

This study was conducted outdoors, with all of the HD-DOT equipment and peripheral technology powered using portable Lithium Iron Phosphate batteries (Tracer Power, South Shields, UK) or portable power banks. An HD-DOT system (LUMO; Gowerlabs Ltd, London, UK) was used to record changes in detected near-infrared light intensity (dual-wavelength light sources emitting at 735 and 850 nm), and subsequently quantify changes in blood oxygenation. Participants each wore an HD-DOT cap consisting of an eight-tile (24 sources and 32 detectors) array over the bilateral auditory cortices and surrounding regions, for a total of 768 possible channels (Figure 1a). Of these, only intra-hemispheric channels had source-detector separations of <40 mm, for a total of 384 (192 channels per-hemisphere) physiologically relevant possible channels. Channels were sampled at a rate of 5Hz.

### Experimental protocol

**Fig. 6:**
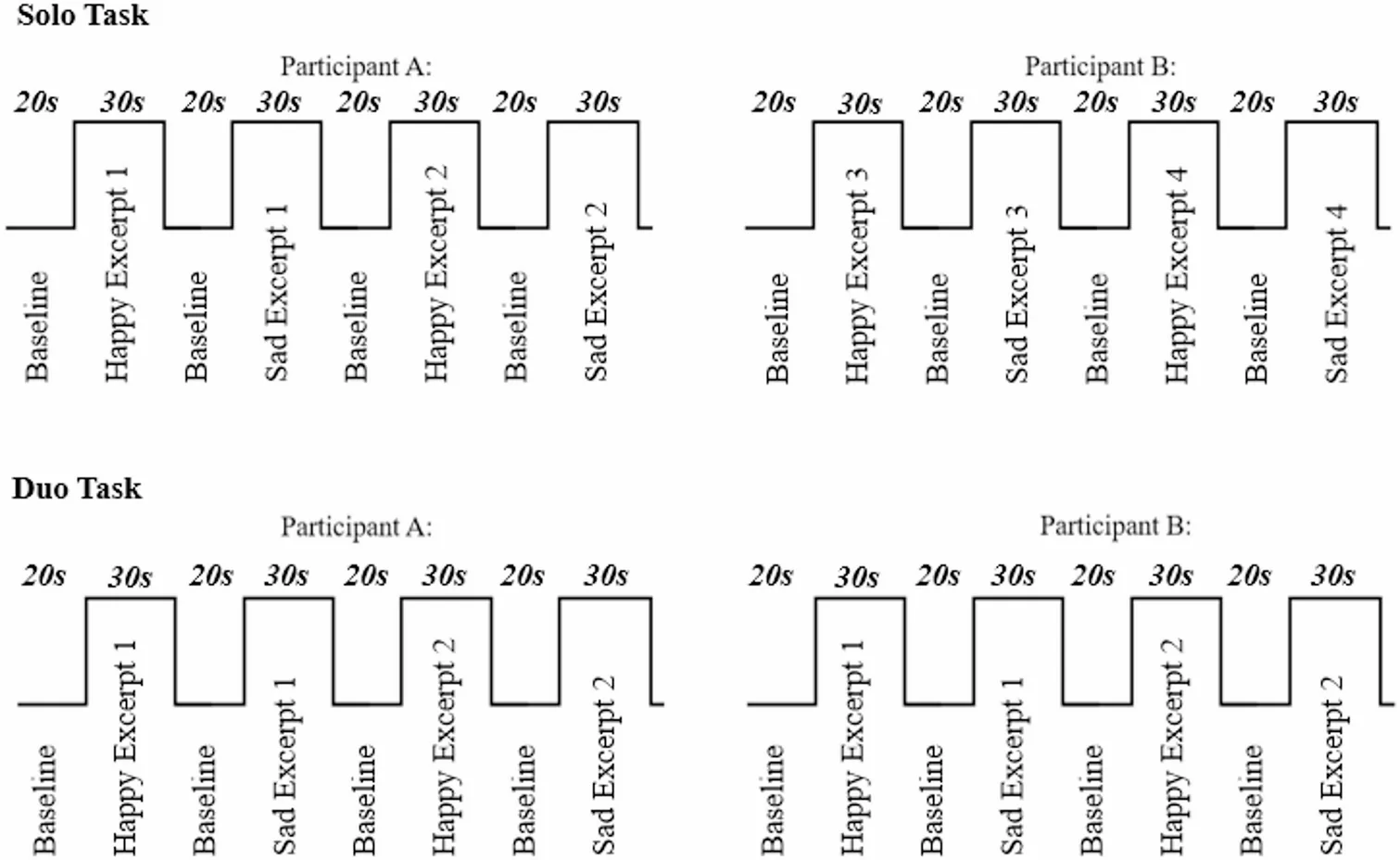
Illustration of experimental protocol. In the solo task (task 1), both participants are listening to different, alternating 30 s excerpts of happy and sad music. In the duo task (task 2), both participants are listening to the same, alternating 30s excerpts of happy and sad music. Participant A listens to the same set of music in both the solo and duo tasks. In both tasks, a 20s baseline period precedes each music stimulus excerpt.

Participants took part in the experiment in pairs, such that each experimental run consisted of two simultaneous HD-DOT recordings. Each participant wore a set of noise-cancelling earphones for the duration of the experiment. The experimental protocol featured two separate tasks, with audio instructions preceding each. These audio instructions described the nature of each task, including the timing of music/rest blocks, and asked participants to try and remember the way each music track made them feel, for the purpose of filling out a written questionnaire following each task. Audio instructions were kept brief (< 1-min) and were followed by an auditory tone to inform participants that the first rest block was beginning. A 2 × 2 within-subjects factorial design was used to investigate the impact of emotional affect and social setting on neural response. In each of the two tasks, participants listened to a total of four separate music tracks. In the solo task, participants were seated opposite each other with a screen in between them to ensure that they could not see each other. Participants were instructed to listen to alternating 30s blocks of film music, with two affective conditions: positive (happy) and negative (sad)^65^. The film music used in this experiment was previously validated for use in assessing music-mediated emotions as part of the study conducted by Eerola and Vuoskoski^65^. These auditory stimulus blocks were preceded by 20s rest blocks to allow the hemodynamic response to return to baseline (Fig. 6). In this task, participants were listening to different music tracks from each other. Upon completion of the solo task, participants were instructed to complete a written questionnaire detailing their qualitative responses to the music (emotional response, familiarity, and liking). In the duo task, the screen separating participants was removed such that they could now see and interact with each other, allowing for two social setting conditions: solo (from the previous task) and duo. The experimental structure for this task remained the same, except that in this case, both participants were listening to the same music tracks as each other. Participant A listened to the same music tracks in the duo task as they did in the solo task, while Participant B was listening to these music tracks for the first time. The purpose of having Participant A listen to the same music across both conditions was to isolate the effect of social context within each pair. Upon completion of the duo task, participants were again instructed to complete a written questionnaire detailing their qualitative responses to the music (emotional response, familiarity, and liking) as well as their perceived feeling of connection to the other listener during the task. Both written questionnaires primarily employed Likert-scale items to assess emotional resonance, liking, and perceived social bonding, while binary (yes/no) questions were included solely to determine participants’ prior familiarity with the music tracks. The wording of questionnaire items was modelled on those used in previous research on music and emotion^25^. To evaluate whether the selected tracks elicited the intended emotional responses (happy or sad), five discrete emotions were included, based on target affect categories established in earlier studies^65^. Further details on the format and phrasing of both written questionnaires (solo and duo tasks) can be found in Supplementary Data.

### Data quality assessment and pre-processing

Optical coupling measures were used to perform a preliminary data quality assessment, ensuring that all included participants demonstrated high optode-scalp contact, which is strongly linked to the signal-to-noise ratio of an fNIRS channel^66^. As such, datasets were included if > 50% of channels in the short separation range (5–10 mm), corresponding to intra-tile channels, met the following channel usability criteria after conversion of raw light intensity data to changes in optical density: (1) mean signal intensity was below 10^11^, (2) source-detector separation was below 100 mm, (3) signal-to-noise ratio was below 12^13,67,68^. All HD-DOT data pre-processing was carried out using the Homer2 fNIRS analysis toolbox in MATLAB^69^. Of the included datasets, individual channels per dataset were rejected according to the aforementioned criteria, as well as if excessive motion was detected (> 25% of the task period impacted by motion artifacts) for any given channel. Motion artifacts were defined as sharp amplitude changes (≥ 0.5) or standard deviation changes (≥ 10) over a 1s time period, with the buffer period ± 1s around each detected motion artifact also being marked as motion burden^70^. Optical data were then pre-processed by first converting optical densities to oxygenated (HbO) and deoxygenated (HbR) hemoglobin concentration changes. A fifth-order Butterworth bandpass filter was used (cut-off: 0.01–0.2 Hz) and local short channel regression (0–12 mm) was performed using the DOT-HUB Toolbox to remove systemic noise from the hemodynamic responses^13,71^. Data were then block-averaged over a 50 s period encompassing the 30s task block with a 10s baseline period pre- and post-stimulus.

### Image reconstruction and parcellation

Prior to statistical analysis, data were min–max normalized to allow for direct comparison of relative changes in hemodynamic response, due to large variations in light intensity across participants. For all participants, a generic mesh model of the head derived from the MNI152 atlas was used as a reasonable estimate^72^. Surface image reconstructions of normalized changes in HbO and HbR concentrations for each participant were generated using Toast++^73^, and group-averaged results were obtained by averaging grey matter surface reconstructions across participants. Using the Schaefer parcellation atlas, individual-level image reconstructions for each condition were parcellated into 400 anatomical regions and registered to 17 functional brain networks, such that each node in the cortical mesh model was assigned to a particular parcel and functional network^39,74^. Measurement sensitivity at each node was calculated during forward modelling for image reconstruction. Nodes demonstrating appropriately high sensitivity to both HbO and HbR changes were classed as sensitive, and parcels for which ≥ 50% of nodes were sensitive were included in the final analysis (see Srinivasan et al.^64^ for a full explanation of parcellation methods). Average HbO and HbR values were calculated for each parcel by taking the mean across all sensitive nodes within the parcel. All subsequent parcel-based analyses (statistical mapping, functional connectivity, and wavelet transform coherence) were carried out in MATLAB R2022b.

### Statistical mapping and functional connectivity analysis

T-statistic maps were produced comparing group-level hemodynamic responses for four contrasts (solo, positive vs solo, negative; solo, positive vs duo, positive; solo, negative vs duo, negative; duo, positive vs duo, negative), where paired sample, parcel-wise t-tests were performed independently to assess areas of significant HbO concentration differences between conditions^75^. This was also done for HbR separately. The entire stimulus block average (30s) was taken as the time window for these t-tests. Corrections using the false discovery rate (FDR) at the *p* < 0.05 level were performed to account for multiple comparisons. Per-participant t-statistic maps were then averaged to produce group-level t-statistic maps. Functional connectivity matrices were also generated for each chromophore (HbO and HbR), and total hemoglobin (HbT = HbO + HbR), via Pearson’s correlation between each parcel pair, across the entire stimulus block average (30s). However, only HbO values are reported in all cases due to an overall higher signal-to-noise ratio across participants and high symmetry between HbO and HbR, particularly with high-density data^76^. Significant parcels were determined following FDR correction at the *p* < 0.05 level. Parcels were grouped according to functional network to assess within- and between-network connectivity for each of the four conditions. Individual-level functional connectivity matrices were then averaged to assess group-level connectivity. Group-level comparison between connectivity matrices was performed following normalization via Fisher’s r-to-z transformation, and the Frobenius distance was used to measure differences between each pair of z-transformed connectivity matrices^77^. Finally, permutation tests were performed for each pair of matrices to assess whether the observed Frobenius distances were statistically significant at the *p* < 0.05 level, by randomly permuting (5000 total iterations) individual rows of one of the connectivity matrices, calculating the new Frobenius distances using each permutation, and comparing against the original (true) Frobenius distances.

### Wavelet transform coherence and hyperscanning analysis

Wavelet transform coherence (WTC) was used to assess the correlation between fNIRS signals originating from paired participants, particularly as WTC can provide information on transient oscillations in non-stationary signals across regions in time–frequency space^78^. Using the wavelet transform, time-series signals were decomposed into time–frequency space by convolving the signal with the scaled and shifted wavelet basis function^79,80^. Here, we have used the analytic Morlet wavelet, as in previous fNIRS studies^81,82^. Coherence maps were generated per parcel for each pair of participants, for both HbO and HbR signals. Average coherence values were determined as the mean coherence within the low frequency band of interest (0.009–0.1 Hz), inside the cone of influence^18,83^. These coherence maps were generated for each of the four conditions and then averaged across all included pairs to create group-level, inter-brain coherence maps. Group-level comparison between coherence maps was performed following normalization via Fisher’s r-to-z transformation, and Euclidean distance was used to assess similarity of coherence patterns between pairs of conditions. To assess whether differences in inter-brain coherence were present across non-paired participants as well, coherence maps were generated for each parcel across every relevant non-pair and subsequently averaged to compare inter-brain coherence between paired and non-paired participants. Non-paired participants refer to all possible participant combinations for which the two participants did not perform the original experiment concurrently but were listening to the same music excerpts as each other. For example, one non-pair would be comprised of Participant A in dyad 1 and Participant A in dyad 2, as they listened to the same music excerpts non-concurrently, but not Participant A in dyad 1 and Participant B in dyad 2, as they were not listening to the same music excerpts. Additionally, parcel-wise, paired t-tests were performed independently to assess whether significant differences in coherence, following FDR correction, were present between paired and non-paired participants for each of the four conditions.

### Statistical analysis of qualitative responses

Participant responses from their written questionnaires (see Experimental protocol) were collated and the mean and standard deviation for each of the numerical rating questions (scale: 0–4) was calculated. All statistical analyses carried out on qualitative responses were conducted in MATLAB R2022b. Table 1 contains details on the specific questions asked and results from the questionnaire evaluation. In particular, we wanted to investigate whether the relationship type for paired participants (friend, family, married/partner, stranger) had a significant effect on the level of connection felt in the duo listening condition. Thus, a Kruskal–Wallis one-way ANOVA was used due to the non-normality of the data and the group size imbalance. Additionally, the correlation between length of relationship (in years) and the level of felt connection was assessed using Spearman’s rank order correlation. Spearman’s rank order correlation was also used to assess the correlation between self-reported level of felt connection and difference in paired and unpaired coherence in the duo listening conditions. For each of the Likert scale questions, a Bonferroni-corrected paired t-test was conducted to assess significant differences in responses between the solo and duo listening conditions (see Supplementary Data for details on questionnaires).

## Supplementary Information

Below is the link to the electronic supplementary material.

Supplementary Material 1

## Data Availability

The datasets generated and analyzed during the current study are available from the corresponding author on reasonable request. This data will be made available publicly via the Openfnirs database ([https://openfnirs.org/data/] (https://openfnirs.org/data)) upon publication.
