# Supplementary material for: Exploring neural correlates of emotional and social listening with high-density diffuse optical tomography in the auditory cortex
Source: Sci Rep. 2026 Aug 26;16:34087. doi: 10.1038/s41598-026-49552-6 (PMC13710171; doi:10.1038/s41598-026-49552-6)
Supplement: Supplementary file 1 [file 41598_2026_49552_MOESM1_ESM.pdf]

Supplementary Material

Exploring neural correlates of emotional and social listening with highdensity diffuse optical tomography in the auditory cortex

Sruthi Srinivasan*, Emilia Butters, Deepshikha Acharya, Ming Hung Hsu, Renee Timmers, Jennifer MacRitchie, Flavia Mancini, and Gemma Bale

* Correspondence: Corresponding Author: ss2814@cam.ac.uk

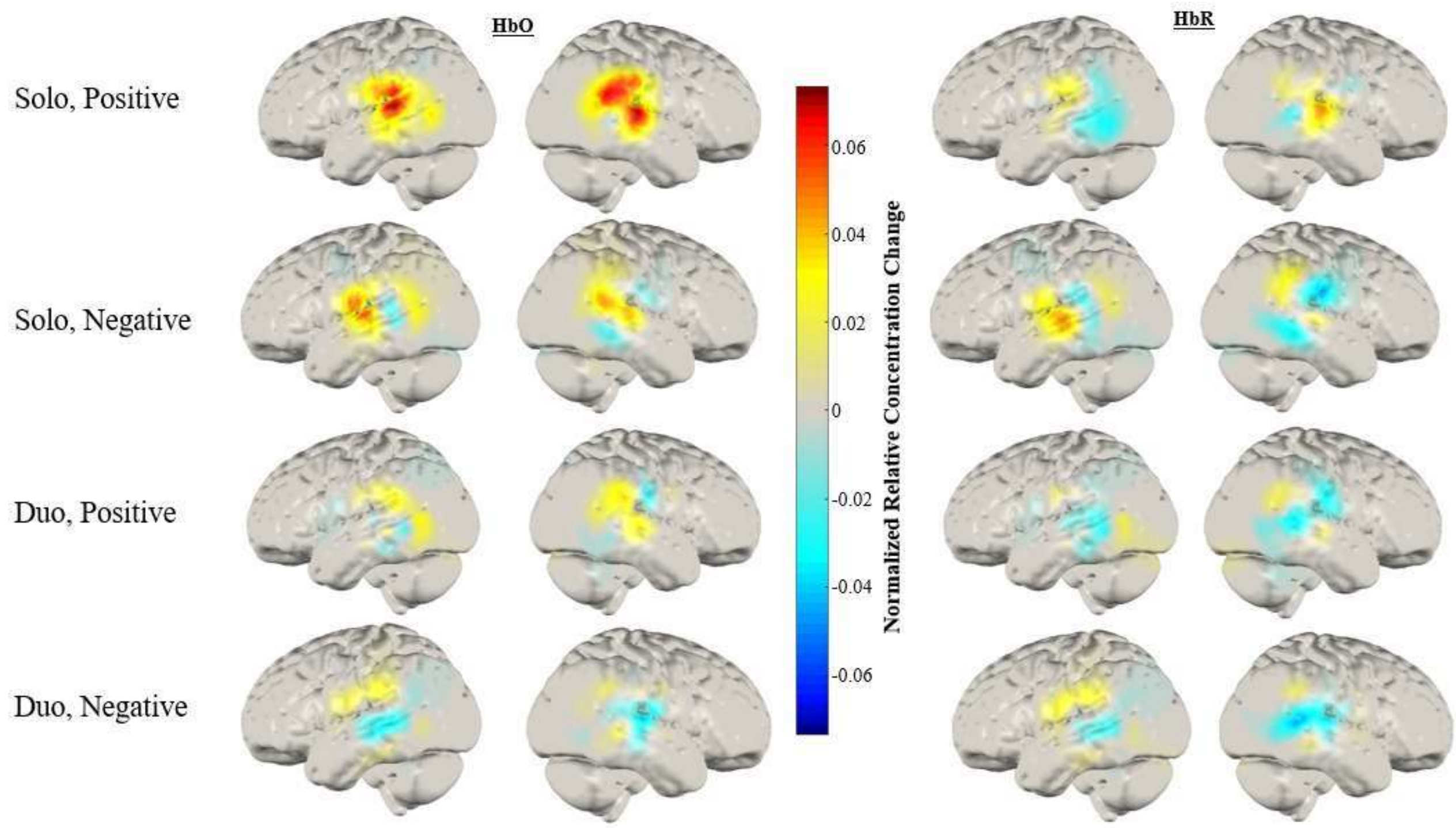

Supplementary Figure S1. Group-level spatial activation maps of normalized relative concentration change for the four conditions (n = 88), shown for HbO (left two columns) and HbR (right two columns). Positive values indicate regions where an increase in the relevant chromophore concentration was observed, while negative values indicate regions where a decrease was observed.

Supplementary Data. Participant questionnaires and demographic information form. Participants were requested to fill out basic information regarding demographic information and level of musical experience prior to the start of the experiment. Written questionnaires were presented to participants twice, once following the solo task and once following the duo task. All participant questionnaires followed the exact format shown below.

Solo condition

What emotion(s) did you feel when listening to the 'happy' music?	Excited	Not at all	A little	Neutral	Somewhat	Very much
	Calm	Not at all	A little	Neutral	Somewhat	Very much
	Scared	Not at all	A little	Neutral	Somewhat	Very much
	Sad	Not at all	A little	Neutral	Somewhat	Very much
	Happy	Not at all	A little	Neutral	Somewhat	Very much

What emotion(s) did you feel when listening to the 'sad' music?	Excited	Not at all	A little	Neutral	Somewhat	Very much
	Calm	Not at all	A little	Neutral	Somewhat	Very much
	Scared	Not at all	A little	Neutral	Somewhat	Very much
	Sad	Not at all	A little	Neutral	Somewhat	Very much
	Happy	Not at all	A little	Neutral	Somewhat	Very much

What emotion(s) did you perceive in the 'happy' music?	Excited	Not at all	A little	Neutral	Somewhat	Very much
	Calm	Not at all	A little	Neutral	Somewhat	Very much
	Scared	Not at all	A little	Neutral	Somewhat	Very much
	Sad	Not at all	A little	Neutral	Somewhat	Very much
	Happy	Not at all	A little	Neutral	Somewhat	Very much

What emotion(s) did you perceive in the 'sad' music?	Excited	Not at all	A little	Neutral	Somewhat	Very much
	Calm	Not at all	A little	Neutral	Somewhat	Very much
	Scared	Not at all	A little	Neutral	Somewhat	Very much
	Sad	Not at all	A little	Neutral	Somewhat	Very much

	Happy	Not at all	A little	Neutral	Somewhat	Very much

How much did you like the 'happy' music?	Not at all	A little	Neutral	Somewhat	Very much
How much did you like the 'sad music?	Not at all	A little	Neutral	Somewhat	Very much
Did you feel connected to the music?	Not at all	A little	Neutral	Somewhat	Very much

Were you familiar with the 'happy' music? Yes / No

Were you familiar with the 'sad music? Yes / No

- -

Dual condition

What emotion(s) did you feel when listening to the 'happy' music?	Excited	Not at all	A little	Neutral	Somewhat	Very much
	Calm	Not at all	A little	Neutral	Somewhat	Very much
	Scared	Not at all	A little	Neutral	Somewhat	Very much
	Sad	Not at all	A little	Neutral	Somewhat	Very much
	Happy	Not at all	A little	Neutral	Somewhat	Very much

What emotion(s) did you feel when listening to the 'sad' music?	Excited	Not at all	A little	Neutral	Somewhat	Very much
	Calm	Not at all	A little	Neutral	Somewhat	Very much
	Scared	Not at all	A little	Neutral	Somewhat	Very much
	Sad	Not at all	A little	Neutral	Somewhat	Very much
	Happy	Not at all	A little	Neutral	Somewhat	Very much

What emotion(s) did you	Excited	Not at all	A little	Neutral	Somewhat	Very much
	Calm	Not at all	A little	Neutral	Somewhat	Very much

perceive in the	Scared	Not at all	A little	Neutral	Somewhat	Very much
	Sad	Not at all	A little	Neutral	Somewhat	Very much

‘happy’ music?	Happy	Not at all	A little	Neutral	Somewhat	Very much

What emotion(s) did you perceive in the ‘sad’ music?	Excited	Not at all	A little	Neutral	Somewhat	Very much
	Calm	Not at all	A little	Neutral	Somewhat	Very much
	Scared	Not at all	A little	Neutral	Somewhat	Very much
	Sad	Not at all	A little	Neutral	Somewhat	Very much
	Happy	Not at all	A little	Neutral	Somewhat	Very much

How much did you like the ‘happy’ music?	Not at all	A little	Neutral	Somewhat	Very much
How much did you like the ‘sad music?	Not at all	A little	Neutral	Somewhat	Very much
Did you feel connected to the music?	Not at all	A little	Neutral	Somewhat	Very much
How strongly connected to the other listener did you feel during the music?	Not at all	A little	Neutral	Somewhat	Very much

Were you familiar with the ‘happy’ music? Yes / No

Were you familiar with the ‘sad music? Yes / No

- -

Contact details

Name: ………………………………………...…………..

Date of birth: ………/………/………

Email: ………………………………………………..……………………………….................…………

Relationship to study partner: ……………………………………………………………..……........……..

Length of relationship with study partner: …………………………………………………........…....…

Which country did you grow up in? ………………………………………………………………..……..

Do you have any musical experience?   None / A little / A lot / Highly experienced

If yes, for how many years have you been playing musical instruments? ………....….. years

How sociable are you feeling today? Not at all / Slightly / Somewhat / Very much

How many alcoholic drinks have you had within the past: 5 hours? ………..   24 hours? ………..

Have you taken any other substances which may affect your task results? Yes / No

Supplementary Table S1. Summary table of participant characteristics. Prior to beginning the experiment, basic demographic information was collected, along with details on the level of prior musical experience and sociability. Participants were asked to provide their date of birth (from which age was calculated), the country they grew up in, their self-assessed level of musical experience (numerical responses ranged from 0 – 3, with 0 corresponding to a qualitative response of 'none' and 3 corresponding to a qualitative response of 'highly experienced'), the number of years they had been playing a musical instrument, and their self-assessed feeling of sociability on the present day (numerical responses ranged from 0 – 3, with 0 corresponding to a qualitative response of 'not at all' and 3 corresponding to a qualitative response of 'very much'). They were also asked to provide information on the number of alcohol drinks they had consumed within set time frames prior to beginning the study. For further details on the wording of questions posed to participants, see the Supplementary Data. Normally distributed variables are presented as mean ± standard deviation, while all non-normally distributed (skewed) variables are presented as median [interquartile range].

**Characteristic**	**Summary Statistics**
Age, years (mean ± standard deviation)	38.16 ± 13.71
Country of Origin (*n*)	160 (total)
United Kingdom	142
Other	18
Level of Musical Experience, scale: 0-3 (mean ± standard deviation)	1.28 ± 0.96
Years of Musical Experience, years (median [IQR])	5 [IQR: 0 – 18]
Level of Sociability, scale: 0-3 (mean ± standard deviation)	2.16 ± 0.73
Number of Alcoholic Drinks in Past 5 Hours (median [IQR])	0 [IQR: 0 – 1]
Number of Alcohol Drinks in Past 24 Hours (median [IQR])	4 [IQR: 1 – 6]
